# Effects of Mobile Phone Use on Driving Performance: An Experimental Study of Workload and Traffic Violations

**DOI:** 10.3390/ijerph18137101

**Published:** 2021-07-02

**Authors:** Carlos A. Catalina Ortega, Miguel A. Mariscal, Wafa Boulagouas, Sixto Herrera, Juan M. Espinosa, Susana García-Herrero

**Affiliations:** 1 Escuela Politécnica Superior, Universidad de Burgos, 09001 Burgos, Spain; cco0001@alu.ubu.es (C.A.C.O.); mariscal@ubu.es (M.A.M.); wafa.boulagouas@umc.edu.dz (W.B.); jespinos@ubu.es (J.M.E.); 2 Departamento de Matemática Aplicada y Ciencias de la Computación, ETS de Ingenieros de Caminos, Canales y Puertos, Universidad de Cantabria, 39005 Santander, Spain; herreras@unican.es

**Keywords:** mobile, phone, distractions, traffic, violations, workload, young, drivers

## Abstract

The use of communication technologies, e.g., mobile phones, has increased dramatically in recent years, and their use among drivers has become a great risk to traffic safety. The present study assessed the workload and road ordinary violations, utilizing driving data collected from 39 young participants who underwent a dual-task while driving a simulator, i.e., respond to a call, text on WhatsApp, and check Instagram. Findings confirmed that there are significant differences in the driving performance of young drivers in terms of vehicle control (i.e., lateral distance and hard shoulder line violations) between distracted and non-distracted drivers. Furthermore, the overall workload score of young drivers increases with the use of their mobile phones while driving. The obtained results contribute to a better understanding of the driving performance of distracted young drivers and thus they could be useful for further improvements to traffic safety strategies.

## 1. Introduction

Traffic accidents account for 1.35 million deaths a year on 2018 and keep being a significant cause of injuries and fatalities [1]. This has led countries all around the world to give the highest priority to improve road safety and devote considerable efforts to manage the injury profiles for traffic accidents and develop a safer road traffic system, such as vehicle safety, road infrastructures improvement, enhancement of drivers’ care, traffic rules and regulations, awareness campaigns, etc.

As a matter of fact, statistics on traffic accidents and related injuries have shown that 80–90% of traffic incidents are caused by drivers’ operational mistakes, errors and misbehaviors, inattention, fatigue, and distraction [2,3,4,5,6].

Although there are numerous potential in-vehicle sources of distraction, extensive research has reported that the use of mobile phones is among the major factors that lead to traffic accidents [7,8,9,10]. In this regard, researchers reported that, in 2015, there were 4.7 billion people using mobile phones and that this number was expected to reach 5.6 billion by 2020 [11]. Furthermore, the development of the telecommunications industry and this increasing number of subscribers would make the use of mobile phones among drivers very common.

Taken together, there is a recent trend and growing interest in technology-based distractions (particularly the use of mobile phones) and a substantial body of research has investigated the risk factors related to their use on the drivers’ performance and road safety. A survey conducted in Australia found that driver distraction contributed to 13.6% of serious traffic crashes [12]. Analogously, in 2019, the Department of Transportation’s National Highway Traffic Safety Administration estimated that distracted driving has claimed around 3142 lives in the US [13]. Moreover, an observational study in the US reported that out of 3265 observed drivers, 32.7% had distracted driving and talking on the phone, and that texting/dialing a phone were among the most frequently observed distractions (i.e., 31.4% and 16.6%, respectively) [14]. As regards predictors for such secondary tasks, an investigation of the observable distractions while driving in the UK found that age emerges as a significant predictor for most of the observed secondary tasks, including mobile phone use [15]. Moreover, this study pointed out that young drivers are more likely to be distracted. Indeed, young people keep using and interacting with their mobile phones too frequently. A naturalistic study found that young people aged between 17–22 years old touch their mobile phones while driving 1.71 times per minute, on average [16]. Similarly, a study involving 254 young participants aged between 17–23 years old found that they touch their mobile phones 1.6 times per minute, and more than half are performed while the vehicle is in motion and half of the screen-touches are to use WhatsApp [17]. Another study involving 114 young people (aged between 17–25 years old) specified that young people have greater use of social media platforms while driving [18]. The descriptive results of this study showed that fully 80.7% of them were chatting and texting, 73.7% were talking on their mobile phones, 53.5% were using Facebook, 41.2% were interacting on Snapchat, and 30.7% were checking their emails.

In Spain, a recent study reported a total of 410,974 traffic accidents occurred in the last four years (2016, 2017, 2018, and 2019). According to this study, these traffic accidents involved 666,504 drivers, of which 8.33% were involved in serious accidents and 12.82% of them were young drivers (under 25 years old) [19]. Moreover, this study found that 4048 of the distracted drivers were using their mobile phones. Similarly, a week-long surveillance campaign carried out in Spain by the DGT (the DGT is the Spanish General Traffic Department) found that 2873 drivers were using their mobile phones while driving [20].

Likewise, out of 10 young people, eight admitted to having distracted driving, and 67% specified that they checked their mobile phones frequently while driving [21]. Another study done in Spain by Linea Directa Foundation in collaboration with the Institute of Traffic and Road Safety (Intras) in 2019, estimated an average mobile phone usage to be 6 h and 48 min and particularly at traffic lights, traffic jams, and when they think “the road is safe” [22]. As regards the use of mobile phones in Spain, it was reported that WhatsApp and Instagram were among the top three applications downloaded and on which the subscribers were more active [23].

It is well established in the literature that distracted driving takes drivers’ eyes off the road, switches their consciousness from driving to other tasks, and results in false perceptions. Indeed, traffic accidents take place when the drivers’ performance is below the required levels for the traffic environment [24]. In this regard, a recent study confirmed that safer driving requires an assessment of driver mental states [25]. Furthermore, the authors have explained that the capacity of humans’ mental resources that could be used to process information received (i.e., mental workload) is limited and the use of mobile phones claims further cognitive resources. Therefore, the margin of the driver’s attentional capacity decreases as long as the amount of information being processed increases. In-depth studies investigated such mental mechanisms have put in light the deployment of numerous approaches and different measures to assess the cognitive load. These approaches could be grouped into four main groups [26]: (i) physiological such as electroencephalogram (EEG), electrocardiography (ECG), galvanic skin response (GSR), and respiration, (ii) eye tracking, (iii) performance-based vehicle speed, and (iv) subjective, e.g., NASA TLX (National Aeronautics and Space Administration Task Load Index) which is the most commonly used assessment tool in the literature

The present study is designed to provide an integrated framework to assess the influence of technology-based distractions (particularly, the use of mobile phones behind the wheel) on driving performance. For this purpose, first, a simulation experiment was conducted using a driving simulator to collect data on driver infractions under the influence of mobile phone distractions. Driving simulators are widely used in human factors, driver behaviors, driver perception, and driver distraction studies as they offer a safe, efficient, and controllable environment. Thus the driving simulator is proved to be a valid substitute for different aspects of real driving experience [27,28,29]. This study was then extended to assess the workload associated with the use of mobile phones while driving. For this purpose, the NASA TLX, which is a well-known subjective multidimensional method, was used to rate six aspects of perceived workload, i.e., mental demand, physical demand, temporal demand, performance, effort, and frustration. In addition to these scales, an overall value of perceived workload was measured as well [30]. Finally, several studies used machine learning techniques in road safety studies to address distracted driving issues [31,32]. In this paper, a tree-based machine learning method was deployed for classification and regression problems.

The rest of the paper is organized as follows: Section 2 describes the design and methodology of the study. Section 3 provides the results, discusses the main findings, summarizes the most relevant weaknesses, and suggests directions for future research. Section 4 concludes the study.

## 2. Background and Related Work

Road safety literature is rich in research and studies that investigated the influence of technology-based distractions on driving performance. A summary of the main findings from 13 selected past studies is given in Table 1.

The selected past studies are reviewed with regard to the objective of the research, the type of the study, and the methodology adopted.

Table 1 shows that mainly three types of research studies are interested in distracted driving due to the use of mobile phones:

(i) survey studies that assess patterns and prevalence of distracted driving and analyze the characteristics of the distracted drivers (e.g., gender and age);
(ii) naturalistic studies that observe behaviors of the drivers and record secondary tasks (in particular, the use of mobile phones) over a period of time; and
(iii) experimental studies that employ driving simulators to design specific driving scenarios, close to a real driving environment, to collect data on the influence of the distractions on the driving performance.

Although survey studies in their different forms (questionnaire, phone interviews, or online), and naturalistic studies are two main approaches that utilize large samples made up of hundreds of subjects to analyze the distracted driving phenomenon, these methods suffer several limitations. First, the data used to conduct such studies involved subjective responses from respondents and interviewees which are, generally, biased and lead to inconsistent outputs. Second, in observational studies, data are collected at one particular location.

Experiment studies, including the present study, are advantageous over other types as they provide a safe and controllable environment for scholars to collect data on distracted driving under specific conditions which are dangerous in the real world or difficult to be reproduced.

Moreover, in comparison of experiment studies of Table 1, and in contrast to survey studies, experiment studies consider typically small samples involving 20–80 participants, and they mainly collect data either on: (i) speed infractions, (ii) lateral control, (iii) lane-changing, and (iv) traffic accidents. Advantageously, the present study is designed to collect several driving performance measures, such as traffic accidents, traffic rules, lateral distance, speeding, and other violations. (Violations studied in this paper will be detailed in Results and Discussions section).

Furthermore, primary methods used to analyze the data collected are the statistical models, for instance, Logistic regressions and Logit models. However, the large number of factors and parameters collected from police reports as regards the traffic accidents challenges the statistical methods to handle all of these variables. Thus, statistical modeling is generally effective in the case of a smaller dataset with fewer attributes, otherwise they end up over-fitting. To address these shortcomings of the statistical models, machine learning techniques have emerged for classification and regression problems. They are more adequate for learning from large datasets with a high number of attributes and observations, for example, Bayesian Networks and Artificial Neural Networks [46], Decision Trees [47], Random Forests [48], etc.

Therefore, in this paper, a tree-based machine learning method (Random Forest) is deployed for classification and regression problems. This method has been previously used to identify when a driver is distracted based on driving behavior data [49], and to establish the relevance of the variables affecting the driving behavior [50], and is found to be a robust and competitive method in both tasks, especially when a limited sample size is available. Despite the differences between the objectives and approaches of the cited studies, the results are consistent with those obtained in this paper, although we extend the analysis to a more general overview of the driving behavior leading to an increment of the traffic infractions.

As part of the underlying causes of distracted driving and resulted impairments, much previous research given in Table 1 used either subjective measures to evaluate the mental workload, such as the NASA TLX and RSME (the Rating Scale Mental Effort), or physiological measures, such as variability in heart rate or changes in brain activity using, for instance, the EEG. Likewise, this study specifically assesses the cognitive workload and efforts of processing auditory and visual information related to the secondary task (i.e., the use of the mobile phone, particularly for calling and texting) using NASA TLX. This design is very convenient to expand the context of previous studies and contribute to the body of literature on technology-based distracted driving.

## 3. Materials and Methods

### 3.1. Participants

In the beginning, a total of 44 subjects participated in the experiment. Nevertheless, five of them were discarded: two participants were eliminated due to age (48 and 56 years old), two others due to missing information on NASA-TLX, and one additional participant was not considered in the study as the recordings of the simulator were corrupted. Finally, only 39 participants successfully completed the experiment. This sample size is consistent with published literature (as discussed in the previous section), and large enough to conduct the study.

**Figure 1:**
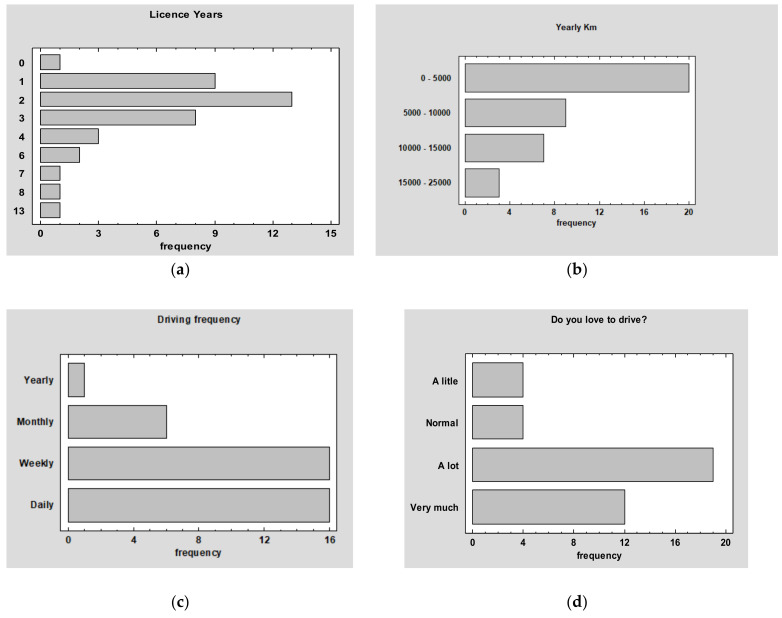
Descriptive of the study sample: (**a**) Frequency of the license years; (**b**) Km driven per year; (**c**) Driving frequency between daily, weekly, monthly and yearly; (**d**) How much the participants love to drive.

The study sample was made up of 12 females and 27 males, aged between 19 and 32 years old (µ = 21.5 and SD = 2.6), and had a valid Spanish driving license. The average number of years since the participants got their driving licenses was 2.84 (SD = 2.38) ((a) of Figure 1). On average, most of the participants drove around 5000 Km per year or less ((b) of Figure 1) and 82% of them drove at least weekly ((c) of Figure 1). Finally, the participants were asked about how much they like to drive and responses showed that most of them liked or liked very much to do so ((d) of Figure 1).

Young people were selected as they use their mobile phones more regardless of road and traffic conditions, are more likely to underestimate the difficulty of driving, and are less likely to understand the risks associated with multitasking [38,51,52,53].

### 3.2. Apparatus

**Figure 2:**
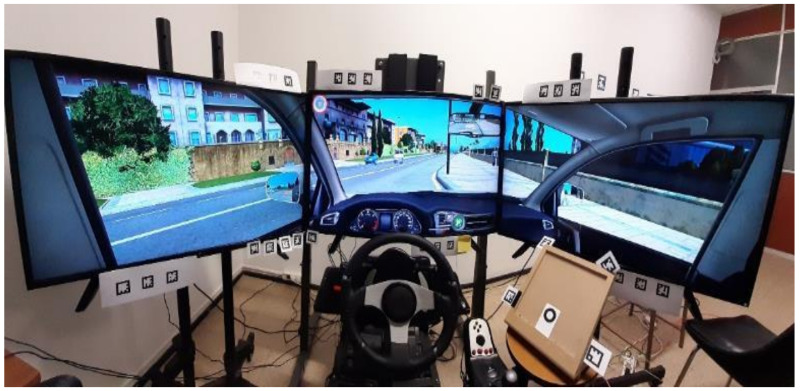
Simulator with three screens used in the study.

For the experiment, an adapted DriveSim simulator located at the University of Burgos, Spain was used. It is a high-quality driving training-based simulator that contains three big screens to yield a high immersive environment, a steering wheel (Logitech G27), pedals, gear lever, and real car controls and signals (ignition key, turn lights, etc.) for more realism (Figure 2).

The simulator screens are 39″ with a 1920 × 1080 resolution each. The simulator can record driving data between 40 and 60 Hz depending on the elements in the environment and the interior of the car, i.e., dashboard and mirrors, as key elements.

The simulator includes artificial intelligent traffic and pedestrian agents to better mimic a real driving experience. A mobile phone was placed to the right of the steering wheel which is commonplace for mobile phones nowadays.

### 3.3. Experimental Design

**Figure 3:**
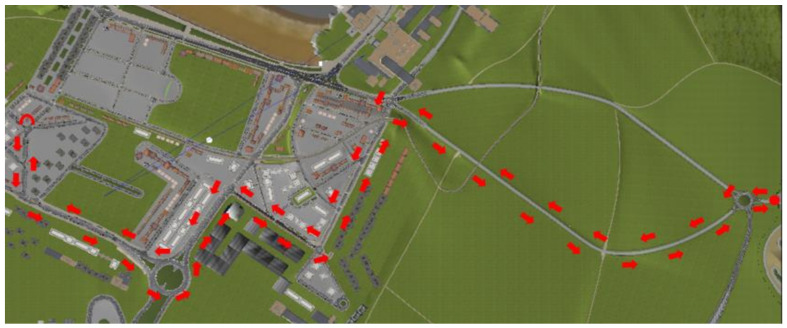
The trajectory of the experiment.

A specific trajectory was created to perform the experiment. It covered rural roads and urban areas and included several roundabouts, stops, traffic lights, and pedestrian crossings (Figure 3). The entire trajectory could be run within about 15 min depending on the traffic and status of the traffic lights. The simulation was configured to have a sunny day with medium traffic and pedestrian density. Both traffic and pedestrians were randomly generated, and the trajectory was complex enough to avoid the learning effect during the laps.

During the experimental phase, two laps were run. The first lap was run with normal conditions in the simulator and no distractions were applied. Into the second lap, the distractions were introduced in the form of several secondary tasks using the mobile phone. The objective was to estimate the effect of mobile phone-related distractions and measure the resulting workload due to multitasking. Accordingly, the participants had to respond to a call, reply to several WhatsApp messages, and use Instagram. These tasks had to be done with a specific mobile phone provided for the experiment placed on the simulator cockpit in a specific position to the right of the steering wheel. The mobile phone had particular contacts on the agenda who should be contacted during the experimental phase.

### 3.4. Experimental Procedure

At their arrival, the participants were introduced to the experimental procedure and asked to sign the bioethical consent. After that, they did a test into the simulator in a free driving scenario for 5–10 min. The aim was to check any potential motion or simulator sickness that could generate a big dropout during the experimental phase. In case the participants felt the motion sickness associated with driving simulators, they were rejected [54,55,56]. Furthermore, the test contributed to making the participants more familiar with the simulator and feel more comfortable during the experiment [57]. Whenever the first test was successfully completed, the participant fulfilled the socio-demographic survey and proceeded with the experiment. Additionally, the participants were informed that they could quit the simulation, at any moment, if they felt any motion sickness or discomfort.

During the experimental phase, two laps were run. In the first lap, the participants got the instructions to drive like they are used to doing in real life with normal conditions on the simulator and no distractions. Following a successful first drive, the participants immediately completed the NASA-TLX.

The NASA-TLX is a well-known tool used to self-evaluate the subjective workload of the volunteer in a task. It rates the overall workload of the task and six other subscales [30]:

- Mental Demand: How much mental and perceptual activity was required? Was the task easy or demanding, simple or complex?
- Physical Demand: How much physical activity was required? Was the task easy or demanding, slack or strenuous?
- Temporal Demand: How much time pressure did you feel because of the pace at which the tasks or task elements occurred? Was the pace slow or rapid?
- Overall Performance: How successful were you in performing the task? How satisfied were you with your performance?
- Effort: How hard did you have to work (mentally and physically) to accomplish your level of performance?
- Frustration Level: How irritated, stressed, and annoyed versus content, relaxed, and complacent did you feel during the task?

Each subscale ranges from “very low” to “very high” respectively, except for the Overall Performance that uses two bipolar descriptors, “success” or “failure”. It also implies the need to fulfill a workload comparison of 15 questions to evaluate the contribution of each dimension to the workload of a specific task.

In the second drive, the participants had to drive and perform several secondary tasks. Nevertheless, they were instructed not to perform the distractions when stopped in red light or similar situations. Particularly, secondary tasks were to respond to a call, replay several WhatsApp messages and later use Instagram.

In case, the participant was not able to perform all the secondary tasks, the data regarding their driving performance were discarded. Immediately, after completing the second drive, the participants completed the NASA-TLX again.

### 3.5. Data Collection

The simulator recorded the telemetry data for each experiment in an SQLite database allowing the collection of a large amount of valuable data related to the simulation. Particularly, telemetry data were associated with the user/simulation and violations. They covered 28 types of records, most of which were focused on the conditions of the vehicle at any given time: speed, control status, accelerations, etc., but also included other valuable data, for instance, the speed limit of the road on which the user was running along. Moreover, the simulator detected inappropriate driving and determined the penalties. In fact, there were up to 87 different conditions in which the simulator registered a penalty, such as exceed the speed limits, flashlights, incorrect use of lights, cross over continuous lines, or go to the side of the road, etc.

As regards data collected from NASA-TLX after the two simulated drives, participants completed physical forms which were later copied into a csv-file (CSV-Comma Separated Value) to be processed easily later.

### 3.6. Study Variables and Data Analysis

Violations were organized in a similar way to a previous study [58] which is consistent with recent research on mobile phone distractions and driving performance [59]. Among 87 types of violations recorded by the simulator, 32 were eliminated as they did not fit the simulation conditions; for instance, switch on the light when driving at night or into the tunnel, park in inadequate places, etc. Out of 55 types of violation left, the participants committed 36 of them, which were grouped into five groups: (i) Lateral Distance: cross over continuous lines, do not respect the distance from the curb, etc.; (ii) Traffic Rules: do not stop at the red traffic light, at a pedestrian crossing or stop sign, incorrect use of turn signals; (iii) Speed: speed violations, i.e., do not respect the speed limits; (iv) Accident: collisions or serious traffic accident; and, finally, (v) Others: drive with the handbrake on, use the clutch incorrectly, etc.

### 3.7. Data Analysis Methods

In order to analyze the main factors contributing to the drivers’ violations, two approaches were considered. On the one hand, the contribution of each feature to the variability if the target variable was estimated by means of an Analysis of Variance (ANOVA). On the other hand, a Random Forest, a tree-based ensemble method, was considered to obtain the importance of each feature to model the target variable [60,61]. Finally, for illustrative purposes, classification and regression trees were used to model the occurrence (Number of violations > 0) and number of violations committed by the participants. In this sense, both regression and classification problems were considered in this work.

#### 3.7.1. Decision Trees

Tree-based methods [62,63,64,65] define a tree as a structure by recursively splitting the features’ space. Each division is obtained by calculating the best predictor split determined by a chosen purity criterion over the target variable. In particular, the Gini Index and the Sum of the Squared residuals (RSS) were considered for classification (Equation (1)) and regression models, respectively. Note that, as a result, a disjoint partition of the features’ space based on the purity criterion is obtained. In the resulting tree, each node corresponds to a test on an attribute (i.e., mobile phone use), each branch corresponds to an attribute value (i.e., mobile use = Yes or No), and each leaf (terminal node) represents a final class/value (i.e., violation = Yes) which is assigned to the subsample fulfilling the different conditions defining the path to reach the leaf node from the top of the tree.

(1) $$\mathrm{GINI}\;=\;1\;-\;\sum_{i=1}^{n}p_{i}^{2}$$

These methods have several well-known advantages and drawbacks. First, the trees have a graphical representation that is easy to be assimilated and interpreted. Compared to other methods, decision trees can be constructed relatively fast and they do not require a very big sample to obtain competitive results [64]. Second, some attributes could not be selected to grow the tree as they are secondary in terms of the increment of the global purity of the partition obtained. For this reason, they can be used as a feature selection in a pre-process for other learning algorithms. Third, they work with both quantitative and qualitative (i.e., discrete) predictors. Finally, for a sufficiently complex (i.e., large or deep) tree, all instances could be correctly classified although this commonly leads to over-fitted models.

#### 3.7.2. Random Forest

Random forest [66,67] is a tree-based bagging (Bootstrap Aggregating) method that constructs N independent trees considering, on the one hand, a random selection of the predictors, and a new sample obtained utilizing a bootstrap over the original one, on the other hand. As a result, N predictions are obtained (one for each tree of the forest) which are combined to obtain the final one.

For the classification and regression, the majority vote class and the mean are commonly used. Note that despite the overfitting of each particular tree, the average of multiple independent trees prevents the overfitting of the random forest. However, the graphical representation of the random forest is not possible and, consequently, its interpretation is more difficult than in the case of the trees. To partially overcome this problem, the importance of each variable, in terms of the error reduction obtained when this feature is chosen in a node, can be obtained as an estimation of the contribution of each feature to the target one.

For this work, the Random Forest has been built considering N = 150 trees, based on cross-validation up to 300 trees, and randomly chosen at most half of the variable features. Based on the results obtained with the tree model, the maximum number of leaf nodes was established in 10, maintaining in some ways the coherence between both approaches.

## 4. Results and Discussions

### 4.1. ANOVA

ANOVA analysis, summarized in Table 2, provides several findings related to the number of violations considering the mobile phone-related distractions.

Results of Table 2 show that data of most of the violations are statistically significant. Indeed, Lateral Distance violations have the highest significance level (*p*-value < 0.001) which is consistent with the literature related to mobile phone distractions and lateral distance violation.

Indeed, outputs of the analysis of a literature review [59] suggested that there are significant differences in the driving performance, in terms of lane position and headway, between distracted and non-distracted drivers. Furthermore, many past studies [68,69,70,71,72] confirmed that several factors that arise from mobile conversations while driving could increase the risk of crashes, for instance, lateral movement, steer speed, steer deviation, and perception-reaction time. While using the mobile phone, the driver stops focusing on the driving task and keeps only one hand on the steering wheel leading to a deterioration in lateral control of the vehicle and potential for a serious traffic accident [73,74].

The simulator used in the experiment does not compute directly the lateral distance. Thus, it is impossible to compute the differences in the lateral distance. Nevertheless, several other violations related to the position of the driver in the road (i.e., cross over hard shoulder line, cross over a continuous line, and do not respect the minimum distance from the curb) were used to estimate the lateral distance.

Two other significant results are found after a deeper analysis of each specific violation. First, the accidents categorized into the simulator as “You have had a serious accident: you have run off the road” is a violation that could be included in the Accident category as well as Lateral Distance. Thus it reinforces previous findings. The second significant violation is related to the speed limit of 20 Km/h.

### 4.2. NASA-TLX Results

ANOVA analysis, summarized in Table 3, provides findings related to the different factors in the workload NASA-TLX considering the mobile phone-related distractions.

Results of Table 3 show that, according to the F values, five dimensions of the NASA-TLX were found to have statistically significant performance differences associated with the use of the mobile phone while driving, namely, Mental Demand, Physical Demand, Effort, Performance, and Overall Workload Score (*p*-value < 0.001).

The results of the analysis of the NASA-TLX of the workload and the influence of the mobile phone distractions on the violations during the two drives are given in Table 4 and Table 5. Results of Table 4 show that the mobile phone-related distractions influence most of the violations and more significantly in the case of Lateral Distance violations and speeding (*p*-value 0.002 and 0.015, respectively). Similarly, results of the NASA-TLX workload in Table 5 show that scores of the overall workload and some subscales are statistically significant. The dimensions Temporal Demand and Frustration of the NASA-TLX did not discard the null hypothesis. In fact, in the case of Temporal Demand, the non-significance could be explained by the fact that in both drives the trajectory run is the same and, consequently, the time necessary to perform the task is quite similar.

Moreover, high significances (*p*-value < 0.001) are found in the case of Mental Demand, Physical Demand, Effort, and the Overall Workload. The participants reported a high workload when using a mobile phone while driving. Furthermore, the dimension Performance of the NASA-TLX is found statistically significant (*p*-value < 0.05), and the participants reported lower scores which could be explained by the fact that the task is much more complex so they, themselves, feel that they drive worse than while driving without using the mobile phone (without distractions).

### 4.3. Random Forest

As the results showed, mobile phone distractions had paramount importance on Lateral Distance Violations. In terms of importance, this variable has the second contribution in the case of the classification problem with nearly 100% together with the Physical Demand. This is also reflected by the results of the ANOVA test (Table 3) in which the contribution of this variable is statistically significant at 99%.

**Figure 4:**
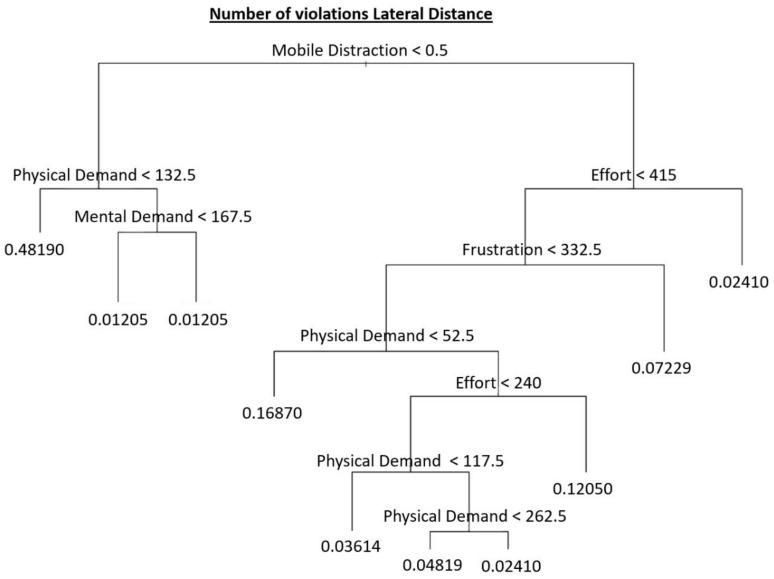
Regression tree related to the number of violations of Lateral Distance.

As regards the regression tree of the Lateral Distance violations, Figure 4 shows the fraction of the sample falling on each end node. Note that the mobile distractions appear in the first nodes reflecting the capability of this variable to isolate homogeneous samples in terms of purity and significant differences in terms of the number of penalizations among both subsamples with and without mobile phone distractions. Moreover, this variable shows paramount importance (~99%) as given by the random forest extending this regression tree.

It is important to note that the rest of the features which contribute to the following nodes lead to deeper branches and correspond to particular cases (the value 0.01205 corresponds to one individual) and are therefore pruned to avoid overfitting issues.

Other interesting results are found as regards one of the specific violations related to Lateral Distance, i.e., the violation “You have crossed over the hard shoulder line”. This means that at least one of the car wheels was over the hard shoulder line of the road.

**Figure 5:**
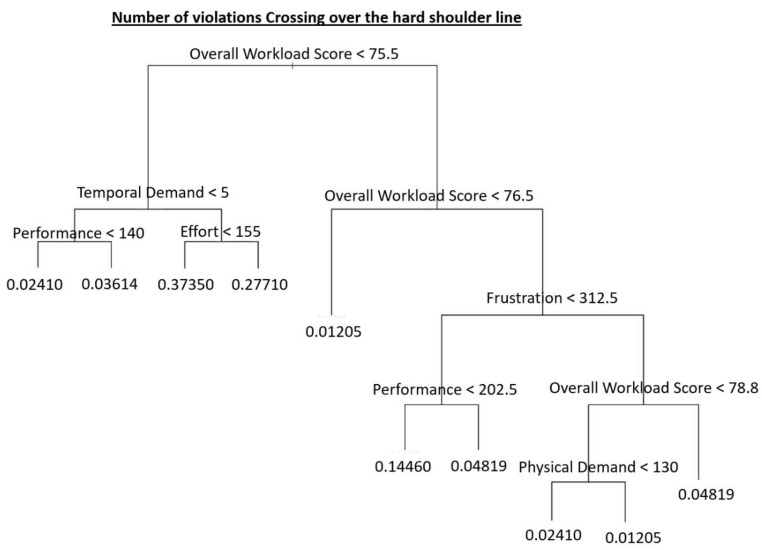
The Regression tree related to crossing over the hard shoulder line violations.

**Figure 6:**
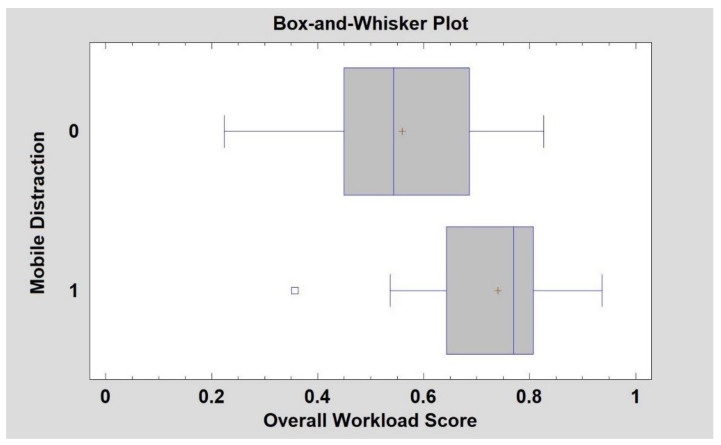
NASA-TLX scores with and without mobile phone distractions.

The first node is the global score of the NASA-TLX that can split the tree with 71% of the cases with a score less than 75.5 (Figure 5). Additionally, it can be noticed that, in the whisker diagram (Figure 6), more than 75% of the values are fewer than 75.5 of the score when there are no mobile phone distractions.

**Figure 7:**
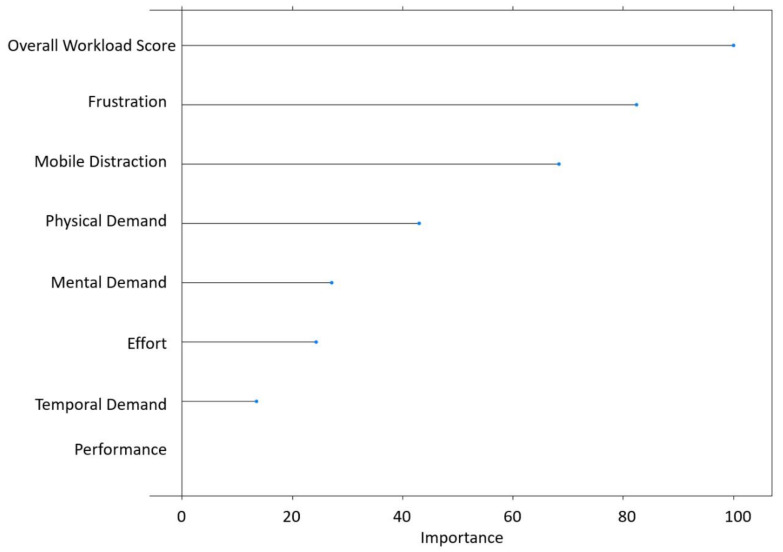
Importance graph related to crossing over the hard shoulder line violations.

As regards the hard shoulder line violations, the results of Figure 5 show that, unlike in the case of Lateral Distance, mobile phone distractions do not appear in the Random Forest. Nevertheless, distractions associated with the use of mobile phones are a key factor in the general forest with an importance score higher than 60% (Figure 7).

Generally, in the Random Forest method, variables compete. Moreover, Overall Workload Score and mobile phone distractions are so related. Consequently, the presence of one variable in the Random Forest is sufficient. In other terms, the presence of mobile distractions in the Random Forest would not provide more information or division of the sample nor explain a residual part. Hence, one variable appears in the Random Forest, the other variable is absent.

The analysis of the importance graphs of the different violations and their related groups shows that the Overall Workload score and mobile phone distractions are found among the two more important factors in the case of 22 specific violations and three groups of violations. Moreover, although these factors do not appear in the Random Forests, their importance is still quite relevant (Table 6).

The findings of the present study come in line with previous research that reported unsafe driving behaviors of young drivers who constitute a high-risk group for traffic accidents, for instance, unsafe driving behaviors of unlicensed young drivers [75,76], risk perception and driving behaviors of young drivers [77,78], and use of mobile phones and infotainment technologies by young drivers while driving [79]. Furthermore, many researchers confirmed that by using their mobile phones, the drivers were more likely to engage in risky driving behaviors, and were less effective in controlling their lane position, managing their brake reaction time, speed, and headway deviation [80,81,82].

In fact, safely driving a motor vehicle requires a permanent monitoring of the road and quick and adequate responses to unexpected changes in the driving environment which depend mainly on the drivers’ manual, visual, and cognitive abilities. However, the use of mobile phones for texting, surfing the web, responding to a call, and checking notifications from social media apps is confirmed to distract the drivers, reduce their attentions, and increase the risk of a crash. In this paper, and using the NASA-TLX, the driver workload was measured and the influence of mobile phone-related distractions was examined. The results obtained confirmed that multitasking (i.e., interacting with the mobile phone while driving) increases the driver’s overall workload. This finding supports the conclusion of many traffic safety studies investigated the driver’s workload in a wide range of driving conditions. For instance, an examination of the effects of road geometry and secondary task modality of the driver’s workload reported that a visually demanding secondary task leads to a significant variance in the driver’s workload [83]. Likewise, a study was conducted to estimate the impact of distractor tasks on the driving performance and driver’s control over the vehicle and workload [84]. Results of this study suggested that the performance of a secondary distractor task increases the workload which influences the variability in steering wheel movements and lane-keeping. Moreover, previous studies used NASA-TLX to investigate the effect of different uses of mobile phones on the drivers’ workload while driving and interestingly found that all the dimensions of the workload are significantly higher in the case of mobile phones use [85,86].

Mobile phone-related distractions are a potential risk to traffic safety and a growing problem that has the biggest impact on driving performance. Even though, the use of mobile phones without a hands-free device is already illegal in many countries, including Spain, eliminating their use seems to be difficult and legislation alone is ineffective in addressing distracted driving. This is because catching offenders is not an easy mission, as with speed. Moreover, the decrease in the use of mobile phones after law enactment does not last long and their use increases immediately following the first decrease.

Therefore, raising public awareness through adoption of behavioral strategies and instruction of positive road safety culture seems to be a promising solution. Indeed, as in the case of seatbelt use and drink-driving, creation of social norms will contribute to changing minds, modifying attitude and behaviors, increasing risk perception, and correcting acceptable risk definition. Particularly, many studies pointed out that young drivers fail to understand the effects of mobile phone use while driving and underestimate the associated risks [87]. Therefore, it is highly important to increase the number of campaigns targeting young drivers through targeted advertising using appropriate communication means and social media platforms, such as Facebook, Instagram, and YouTube.

Furthermore, a recent cross-sectional study conducted in Spain pointed out that older drivers affect their children’s driving attitudes and behaviors. Therefore, another trend to improving traffic safety education consists in enhancing parents’ road behaviors to influence positively the way their children, i.e., young drivers, perceive traffic safety and behave at the wheel [88].

Another equally important awareness program to minimize technology-based distractions, especially among young drivers, is the use of video game simulation methods. Researchers who applied these methods reported a reduction in mobile phone use among participants in the simulation experiment and confirmed that video game simulations were practical and cost-effective programs for training young drivers [89].

In terms of limitations, the nature of the present study, which used a driving simulator, considered only one driving scenario (i.e., a sunny day with medium traffic and pedestrian density), consequently, the results could not capture all possible violations in a real-driving environment.

Another possible limitation is the fact that the study sample was not representative and contained more males than females, as a result, the differences between the driving performance of distracted females and distracted male drivers (i.e., the influence of gender) were not investigated.

Further studies could move towards assessing the influence of technology-based distractions on driving performance in different conditions and consider analyzing the gender effects.

In this study we focused on young drivers. We also recommend studying the effect of mobile phone use among other age groups on driving behaviors.

## 5. Conclusions

The explosive development of communication means and infotainment technologies affects the driving performance of young drivers, particularly. Indeed, the use of mobile phones while driving has been identified among principal contributors to traffic accidents.

This study analyzed, experimentally, changes in the workload and vehicle control (such as lateral distance and hard shoulder line violations) of distracted and non-distracted young drivers. The findings of the present study confirmed the impairments associated with the use of mobile phones among young drivers leading to poor control of the vehicle. Thus, decision-makers need to consider raising awareness of young drivers of mobile phone use risk behind the wheel and encourage the implementation of active measures to mitigate this risk. Actually, some mobile phones are starting to have a driving mode on their operating systems that, when detecting that the user is driving, turns their phone operation into easy-use, or restricted, mode. Moreover, the incorporation of the eye-tracker system as a passive safety measure in vehicles would notify drivers whenever they engage in other activities which distract their attention from the roadway. Meanwhile, educational campaigns targeting young drivers on mobile phone use while driving must be reinforced to minimize risks.

## Figures and Tables

**Table 1:** Summary of similar past studies.

Paper	Research Type	Dataset Characteristics		Data Collected		Instruments		Distractions		Random Forest
		Sample Size	Age Group	Workload	Behavioral Data	Workload Assessment Tool	Data Collection Tool	Mobile Phone Distractions	Other Distractions	
[33]	E	20 20	25–45 > 65	X	Crash rate Acceleration Lane position	NASA TLX	Simulator	-	Challenging road events	-
[34]	N	11 9	13–34 39–51	X	Task-related interior glances	NASA TLX	-	Texting	-	-
[35]	E	20	20 (SD 3.1)	X	Collision; Aggressive driving Traffic rules violations Lane keeping	NASA TLX	Simulator	-	Affective states	-
[36]	E	16 16 20	19.2 (SD 2.3) 19.1 (SD 1.3) 19.9 (SD 1.1)	X	Lateral position	NASA TLX	Simulator	-	Road conditions	-
[37]	E	50	18–59	-	Lane excursions	-	Simulator	Texting	-	-
[38]	E	34	18–30	X	Speed Variance of lane position	NASA TLX	Simulator	Calling	Road conditions	
[39]	S	200	39.5 (SD 10.2)	X	Driving accidents Human errors	NASA TLX and CFQ cognitive failure questionnaire	-	-	-	-
[40]	E	25 24	22.12 (SD 2.45) 37.62 (SD 7.22)	X	Longitudinal and lateral controls	-	Simulator	Texting and calling	-	-
[41]	E	34	Male: 32.5 (SD 5.38) Female: 30.46 (SD 4.2)	X	Car-following	EEG	Simulator	Calling	-	-
[42]	S	475 232	≤30 >30	X	Situation awareness Driving performance	SWAT	-	-	High altitude environment	-
[43]	N	20	57.8 (SD 2.7)	X	Speed variability Reaction time Number of traffic violations	NASA TLX & EEG	-	-	Three complexity levels of the situation	-
[44]	E	18 18 18 18	18–25 31–40 55–65 70–80	-	Braking responses	EEG	Simulator	-	Acoustic and visual distraction stimuli	-
[45]	E	41	18–61	-	Gap acceptance at intersections Intersection crossing completion time	-	Simulator	Texting	-	-

E: Experimental, N: Naturalistic (observational), S: Survey.

**Table 2:** ANOVA test for the number of violations.

Violations	Sum Sq	Mean Sq	F Value	Pr (>F)
Lateral Distance	73.220	73.221	14.169	0.000 ***
—Crossing over a hard shoulder line	12.732	12.733	6.138	0.015 *
—Crossing over a solid line	13.190	13.190	13.486	0.000 ***
Traffic Rules	3.800	3.800	0.011	0.915
Speed	128.700	128.707	1.526	0.220
—Speed limit 20 Km/h	6.222	6.222	6.773	0.011 *
Accident	2.101	2.101	1.170	0.283
—Accident out of the road	0.609	0.609	4.321	0.041 *
Others	2.300	2.297	0.115	0.736

* *p*-value < 0.05 *** *p*-value < 0.001.

**Table 3:** ANOVA Test summary of NASA-TLX sources of workload.

NASA TLX	Sum Sq	Mean Sq	F Value	Pr (>F)
Mental Demand	155,478	155,478	13,365	0.000 ***
Physical Demand	84,298	84,298	12,794	0.001 ***
Temporal Demand	22,908	22,908	18,464	0.178
Effort	134,221	134,221	14,336	0.000 ***
Performance	33,466	33,466	46,122	0.035 *
Frustration level	39,524	39,524	23,094	0.133
Overall Workload Score	6596	6596	38,997	1.851 × 10^−8^ ***

* *p*-value < 0.05; and *** *p*-value < 0.001.

**Table 4:** Comparison of driving performance of the drivers during the two drives.

	No Distractions			Distractions			Differences				
Variable	µ	SD	C.I. (95%)	µ	SD	C.I. (95%)	µ	SD	C.I. (95%)	T-Student	*p*-Value
Lateral Distance	2.54	2.10	(1.86; 3.22)	4.28	2.70	(3.40; 5.16)	−1.74	0.55	(−2.84; −0.65)	−3.18	0.00
LD: crossing over a hard shoulder line	1.13	1.15	(0.76; 1.50)	1.97	1.69	(1.43; 2.52)	−0.85	0.33	(−1.50; −0.19)	−2.58	0.01
LD: crossing over a solid line	0.69	0.69	(0.47; 0.92)	1.49	1.21	(1.10; 1.88)	−0.80	0.22	(−1.24; −0.35)	−3.56	0.00
Speed limit 20 Km/h	1.46	0.94	(1.16; 1.77)	2	0.97	(1.68; 2.32)	−0.54	0.22	(−0.97; −0.11)	−2.48	0.02
Accident out of the road	0.03	0.1	(−0.03; 0.08)	0.21	0.52	(0.04; 0.37)	−0.18	0.09	(−0.35; −0.01)	−2.05	0.04

µ: Mean, SD: Standard Deviation, and C.I: Confidence Interval.

**Table 5:** Comparison of the results of NASA-TLX dimensions during the two drives.

	No Distractions			Distractions			Differences				
Variable	µ	SD	C.I. (95%)	µ	SD	C.I. (95%)	µ	SD	C.I. (95%)	T-Student	*p*-Value
Mental Demand	238.20	97.12	(206.72; 269.69)	326.03	115.71	(288.52; 363.54)	−87.82	24.19	(−135.99; −39.64)	−3.63	0.001
Physical Demand	35.64	45.91	(20.76; 50.52)	102.18	106.50	(67.66; 136.7)	−66.54	18.57	(−103.52; −29.55)	−3.58	0.001
Effort	144.49	83.07	(117.56; 171.41)	222.31	109.21	(186.90; 257.71)	−77.82	21.97	(−121.58; −34.06)	−3.54	0.001
Performance	152.82	96.61	(121.51; 184.14)	326.03	115.71	(288.52; 363.54)	−87.82	24.19	(−135.99; −39.64)	−3.63	0.001
Overall Workload Score	0.56	97.12	(206.72; 269.69)	102.18	106.50	(67.66; 136.7)	−66.54	18.57	(−103.52; −29.55)	−3.58	0.001

µ: Mean, SD: Standard Deviation, and C.I: Confidence Interval.

**Table 6:** Summary of violations in which the importance of the Overall Workload Score and mobile phone distractions is among the three more relevant.

Violations	Overall Workload Importance	Mobile Phone Distractions Importance
Group of Lateral Distance Violations		2
Group of Speed Violations		2
Group of Other Violations	2	
Go through the amber light	1	
Do not stop at a red signal light		2
Failure to yield correctly	1	
Do not stop at a stop signal	1	
Drive in a forbidden direction	1	
Cross over a solid line	1	
Do not stop at a pedestrian crossing		2
Stopover an intersection with yellow crossing lines	1	2
Do not respect the minimum distance from the curb	1	
Exceed the speed limit of 20 km/h		2
Exceed the speed limit of 40 km/h	1	2
Exceed the speed limit of 70 km/h	2	
Exceed the speed limit of 90 km/h	2	1
Exceed the speed limit of 100 km/h	1	
Bump another vehicle	2	
Bump an object		2
Hit another vehicle	1	
Serious accident: you have run off the road		2
Hit a cyclist	2	
Stall the vehicle	1	
Do not fasten the seat belt	1	
Incorrectly use the clutch		2
